# Association of levels of antibodies against citrullinated cyclic peptides and citrullinated α-enolase in chronic and aggressive periodontitis as a risk factor of Rheumatoid arthritis: a case control study

**DOI:** 10.1186/s12967-015-0625-7

**Published:** 2015-08-29

**Authors:** Stefan Reichert, Wolfgang Schlumberger, Cornelia Dähnrich, Nora Hornig, Wolfgang Altermann, Hans-Günter Schaller, Susanne Schulz

**Affiliations:** Department of Operative Dentistry and Periodontology, Martin-Luther University Halle-Wittenberg, Große Steinstrasse 19, 06108 Halle (Saale), Germany; EUROIMMUN AG, Lubeck, Germany; HLA Laboratory (Ghatt), Martin-Luther University Halle-Wittenberg, Halle, Germany

**Keywords:** Periodontitis, Rheumatoid arthritis, Cyclic citrullinated peptides, Citrullinated α-enolase, *P. gingivalis*

## Abstract

**Background:**

Periodontal disease could be a risk factor for rheumatoid arthritis (RA). It is assumed that the bacterial strain *Porphyromonas gingivalis* mediates citrullination of host peptides and thereby the generation of RA-associated autoantibodies in genetically predisposed individuals. For that reason non-RA individuals who suffered from generalized aggressive (GAgP, N = 51) and generalized chronic periodontitis (GChP, N = 50) were investigated regarding the occurrence of antibodies against citrullinated cyclic peptides (anti-CCP) and citrullinated α-enolase peptide-1 (anti-CEP-1) in comparison to non-RA non-periodontitis controls (N = 89). Furthermore, putative associations between infections with five periodontopathic bacteria or expression of certain human leucocyte antigens (HLA) to these autoantibodies were investigated.

**Methods:**

The presence of anti-CCP and anti-CEP-1 in plasma samples was conducted with enzyme linked immunosorbent assay. Subgingival plaque specimens were taken from the deepest pocket of each quadrant and pooled. For detection of DNA of five periodontopathic bacteria PCR with sequence specific oligonucleotides was carried out. Low resolution HLA typing was carried out with PCR with sequence specific primers. Differences between patients and controls were assessed using Chi square test with Yates correction or Fisher`s exact test if the expected number n in one group was <5.

**Results:**

Two patients with GAgP (3.9 %), no patient with GChP and two controls (2.2 %, p_Fisher_ = 0.662) were positive for anti-CEP-1 whereas no study participant was anti-CCP positive. Individuals with *P. gingivalis* were slightly more often anti-CEP-1 positive in comparison to individuals without *P. gingivalis* (3.2 vs. 1.1 %, p_Fisher_ = 0.366). Carrier of HLA-DQB1*06 or the HLA combination DRB1*13; DRB3*; DQB1*06 were slightly more anti-CEP-1 positive (6.1 and 4.3 %) than no carriers (0.7 and 0 %, p_Fisher_ 0.053).

**Conclusions:**

GAgP and GChP and the presence of periodontopathic bacteria are not associated with an increased risk for occurrence of anti-CCP and anti-CEP-1 autoantibodies. The putative relationship between periodontitis and RA should be investigated in further studies.

## Background

The etiology and pathophysiological mechanisms of rheumatoid arthritis (RA) are intensively discussed (overview: [1–3]). It is assumed that an environmental agent triggers an autoimmune reaction in a genetically predisposed individual [4] with subsequent synovial inflammation and cartilage bone destruction [5]. This hypothesis is supported by the discovery of citrulline-specific autoimmunity with generation of anti-citrullinated peptide/protein antibodies (ACPA) in patients with RA [6, 7]. Citrullination is the post-translational conversion of peptidylarginine to peptidylcitrulline wherein the terminal positively charged amino group of the arginine is cleaved. Citrullination could lead to changes in tertiary structure of the protein which render the protein more susceptible to degradation [8]. While citrullinated peptides play a role in a lot of physiological processes such as epithelial terminal differentiation, gene expression regulation and apoptosis, the generation of ACPAs appears to be specific for RA. Therefore, tests for the detection of antibodies against mutated citrullinated vimentin (MCV) or cyclic citrullinated peptides (CCP) are valuable tools especially for the early diagnosis of RA [9].

Citrullination is catalysed by a family of calcium-dependent peptidylarginine deiminases (PAD). In humans, five types (PAD-1 to 4 and PAD-6) have been described [10] but only PAD-2 and PAD-4 are found in the synovial tissues of patients with RA and those with other arthritis forms [11].

RA is genetically associated with certain human leucocyte antigen (HLA) class II molecules (HLA-DRB1*0101, -DRB1*0401 and –DRB1*0404) that contain a shared epitope. It is assumed that these HLA molecules may influence disease pathogenesis by selectively binding arthritogenic peptides for presentation to autoreactive CD4^+^ T cells [12]. In binding assays it was shown that citrullination of vimentin significantly increases peptide–binding affinity to HLA-DRB1*0101, -DRB1*0401 and -DRB1*0404 in comparison to non-citrullinated vimentin and leads to the activation of CD4^+^ T cells and subsequently to generation of autoantibodies against citrullinated peptides. In HLA alleles which were not associated with RA such as HLA-DRB1*0802, -DRB1*1101 and -DRB1*1302 such differences in binding affinity could not be detected [13]. These results suggest that initiation of autoimmune response to citrullinated peptides is HLA-restricted.

The discovery that a major bacterium of periodontal disease, *Porphyromonas gingivalis*, expresses its own unique PAD called PPAD [14] supported the hypotheses that periodontitis and periodontopathogens may contribute to the etiology of RA [4]. It has been shown that PPAD is able to citrullinate bacterial as well as host peptides such as fibrinogen and α-enolase [15]. It is feasible that ACPA can be generated in the gingiva of patients with periodontal disease as a consequence of *P. gingivalis*-induced citrullination of bacterial or/and human proteins by PPAD, human PAD, or both. These ACPAs may be cross-reactive with synovial citrullinated peptides which were formed after a second inflammatory event in the joint [4]. Indeed, PAD-2 and PAD-4 as well as citrullinated peptides were detected in human gingiva and there was an association between inflammation and expression of these peptides. Moreover, antibodies against CCP were found mostly in gingival crevicular fluid of patients with periodontitis in comparison to non-periodontitis individuals [16].

Six cross-sectional studies investigated putative associations between periodontal disease and levels of circulating ACPA. Two case–control studies demonstrated a slightly increased percentage of patients with aggressive periodontitis (AgP) who were anti-CCP positive (7.4 or 8 %) when compared to controls (0 %) [17, 18]. No anti-CCP antibodies were found in patients with chronic periodontitis (ChP) or gingivitis [18]. However, these results were not statistically confirmed. In a third case–control study a significantly higher percentage of anti-CCP-positivity was observed among patients with periodontitis (8 %) compared to healthy controls (0 %, p < 0.001). Additionally, the periodontitis group harboured significantly higher levels for anti-citrullinated α-enolase peptide 1 (anti-CEP-1) and anti-arginine containing enolase control peptide (anti-REP-1). Periodontitis patients who were positive for *P. gingivalis* in subgingival plaque showed a significantly increased level for anti-CCP in comparison to *P. gingivalis*-negative patients. In contrast, both anti-CEP-1 and anti-REP-1 levels were similar irrespective of the presence of *P. gingivalis* [19]. A fourth case–control study demonstrated in a group of patients with moderate to advanced periodontitis more individuals who were anti-CEP-1 positive when compared to non-periodontitis controls (12 vs. 3 %, for age, gender, smoking adjusted Odds ratio: 1.65 95 % CI 0.37–7.5, p = 0.5) [5]. However, the number of individuals who were anti-CCP positive was not different (1 vs. 1 %). In a recently published cohort analysis among a Japanese healthy population slightly positive associations between missing teeth (adjusted OR = 1.04 95 % CI 1.02–1.06, p = 0.024), the Community Periodontal Index (adjusted OR = 1.35 95 % CI 1.15–1.48, p = 0.0042), loss of attachment (adjusted OR = 1.18 95 % CI 1.01–1.37, p = 0.037) to ACPA positivity was shown [20]. Finally a sixth case–control study reported about an association between anti-CCP level and alveolar bone loss >20 % (p = 0.03) in patients with RA in comparison to patients with osteoarthritis [21].

The results of the previous studies suggest that periodontitis and/or the infection with *P. gingivalis* may be associated with the level of circulating ACPA. Beyond that the following questions were of particular interest: At first, the main periodontitis forms AgP and ChP are different in their onset, course, and possibly in their underlying genetic background. Therefore, we decided to examine whether AgP and ChP are different in ACPA levels. Secondly, we were interested in investigating whether apart from *P. gingivalis* other main periodontopathic bacteria were associated to ACPA. Thirdly, because generation of ACPA is HLA-restricted, it is important to investigate whether certain RA-related HLA-alleles are associated to ACPA in patients with periodontitis.

Therefore the first aim of this study was to investigate the level of ACPA in patients with generalized AgP (GAgP) and generalized ChP (GChP) in comparison to controls without periodontitis. Secondly, we examined whether the formation of ACPA was also associated with the presence of *Aggregatibacter actinomycetemcomitans*, *Prevotella intermedia*, *Tannerella forsythia*, and *Treponema denticola* in subgingival plaque specimens. Finally we aimed at investigating whether ACPA formation was associated with the individual expression of RA-related HLA alleles. For the analysis of ACPA, we include anti-CCP and anti-CEP-1. Anti-CCP antibodies are highly specific for RA (92–98 % vs. asymptomatic blood donors 0.5 %) and have important relevance for early diagnosis of the disease [22]. Citrullination of α-enolase was found to be related to *P. gingivalis* strains [15]. Anti-CEP-1 antibodies were observed in 37–62 % of patients with RA (healthy controls 2 %) [23].

## Methods

### Study population and clinical investigations

The study was approved by the local ethics committee and was carried out in accordance with the ethical guidelines of the “Declaration of Helsinki” 1975 and its amendment in “Tokyo and Venice”. The study was performed at the Department of Operative Dentistry and Periodontology of the Martin-Luther University Halle-Wittenberg. The patients and controls were recruited from June 1996 to May 2014 as previously published [24]. Therefore, the inclusion and exclusion criteria are only briefly described.

Overall, 51 patients with GAgP, 50 patients with GChP and 89 individuals without periodontitis were included. All individuals were unrelated Germans of Caucasian descent. They had no known medical or general health conditions that might profoundly contribute to development of periodontitis. For instance, patients with RA, diabetes mellitus, Morbus Crohn, coronary heart disease, patients who took regularly anti-inflammatory drugs or developed gingival overgrowth due to specific drugs such as anti-epileptics, calcium-channel blockers, cyclosporine and pregnant women were not included. Moreover, the use of antibiotics or subgingival scaling and root planing 6 months before the beginning of clinical and microbial examination led to exclusion.

The patients were assessed as previously described [24] in accordance with the new classification system of periodontal diseases [25].

Patients with GChP were selected if they showed a clinical attachment loss (AL) ≥4 mm in at least 30 % of the teeth. The amount of destruction of the periodontal tissues was commensurate with the presence of microbial plaque including subgingival calculus and other local predisposing factors. In the radiographs an even horizontal bone reduction was frequently visible. Angular bony defects were rare.

Patients with GAgP were included only in case of evidence (dental history and/or radiographs) that the age of onset was <35 years. At the time of the clinical diagnosis they had an AL ≥4 mm of at least 30 % of the teeth. In order to exclude a localized aggressive periodontitis at least three teeth had to be affected which were not first molars or incisors. Conversely to GChP, the severity of destruction of periodontal tissues was inconsistent with the amount of microbial deposits. In the radiographs angular bony defects were often visible.

Periodontitis-free individuals were included only in case they were not younger than 30 years and did not have any clinical attachment loss, i.e., PD values ≤3.5 mm and no gingival recession due to periodontitis. Individuals with vestibular AL values of >3.5 mm caused by traumatic tooth brushing or former orthodontic therapy were not considered as cases of periodontitis. AL due to overhanging subgingival restorations or endodontic lesions on single teeth did not lead to exclusion, if all other teeth had no signs of a periodontitis. Moreover, pseudo pockets over the cemento–enamel junction with a pocket depth of >3.5 mm on the last molars were not considered as periodontitis case.

During anamnesis the periodontitis patients were asked about the onset and course of the diseases. Patients with GAgP often reported fast progression of the disease and episodes of acute gingivitis, abscess formation, tooth loosening or dental loss caused by tooth loosening, and many unsuccessful attempts to heal the disease. Additionally, all participants were questioned regarding their smoking status. A person who smoked at least one cigarette per day was considered a current smoker. A smoker who had no smoked for at least 1 year was defined as past smoker. The clinical assessment included the determination of the approximal plaque index (API %) [26], the percentage of teeth with bleeding on probing (BOP %), PD (mm) as a distance between the gingival margin and the bottom of the pocket, and clinical AL (mm) as a distance between the cemento-enamel junction and the bottom of the pocket.

For each tooth both the maximal values for PD and AL were derived by measuring six sites around each tooth. In order to determine the extent of periodontitis in one person, the percentage of teeth with AL of more than 6 mm was recorded. The demographic and clinical data are given in Table 1.

**Table 1:** Clinical characteristics of the patient groups in comparison to the control group (no periodontitis)

	Generalized aggressive periodontitis (N = 51)	Generalized chronic periodontitis (N = 50)	No periodontitis (N = 89)
Clinical parameters			
Age median (25th/75th percentiles)	42.0 (36.0/49.0)*	48.0 (43.0/56.3)*	44.0 (39.0/56.0)
Females, n (%)	29 (56,.9)	31 (62.0)	45 (50.6)
Current smoker, n (%)	17 (33.3)	13 (27.1)	19 (21.3)
Current and past smokers, n (%)	26 (51.0)	23 (47.9)	33 (37.1)
Approximal plaque index %	57.0 (32.0/71.0)	56.0 (36.8/87.0)	43.0 (31.4/61.0)
Bleeding on probing (%) median (25^th^/75^th^ percentiles)	88.0 (75.0/100.0)*	79.5 (61.3/93.3)*	44.8 (30.0/63.0)
Probing depth (mm) median (25th/75th percentiles)	5.3 (4.6/6.6)*	4.9 (4.2/6.1)*	2.6 (2.2/2.8)
Clinical attachment loss (mm) median (25th/75th percentiles)	6.2 (5.7/7.3)*	5.8 (4.7/6.8)*	2.9 (2.6/6.3)
Teeth with AL >6 mm (%) median (25th/75th percentiles)	46.4 (34.5/60.7)*	32.3 (14.1/57.4)*	0
Missing teeth median (25th/75th percentiles)	2.0 (0/4.0)	3.0 (1.0/6.0)	2.0 (0/4.0)
Individual occurrence of periodontal bacteria in subgingival pockets			
*A. actinomycetemcomitans*, n (%)	18 (35.3)*	20 (40.8)*	15 (17.0)
*P. gingivalis*, n (%)	37 (72.5)*	37 (75.5)*	19 (21.6)
*P. intermedia*, n (%)	28 (54.9)*	30 (61.2)*	27 (30.7)
*T. forsythia*, n (%)	43 (84.3)	47 (95.9)*	60 (68.2)
*T. denticola*, n (%)	43 (84.3)*	47 (95.9)*	55 (62.5)

*AL* clinical attachment loss.

* p ≤ 0.05 in comparison with the control group.

### Molecular assessment of periodontopathic bacteria

After removal of supragingival plaque and relative drying, microbial samples were taken from the deepest pocket of each quadrant by insertion of a sterile paper point for 20 s. All bacterial plaque samples of each individual were pooled in one tube.

A molecular biological test (Micro-Ident test, Hain Lifescience, Nehren, Germany) was used for identification of the five periodontogenic bacterial species. The whole procedure was divided into three steps, i.e. DNA isolation, multiplex amplification with biotinylated primers, and reverse hybridization.

The preparation of bacterial DNA was carried out using a commercial DNA kit (QIAamp DNA Mini Kit; Qiagen, Hilden, Germany) according to the producer’s manual. For specific amplification of bacterial DNA 17.5 µl Primer-Nucleotide-Mix, 2.5 µl 10× polymerase incubation buffer, 1 µl MgCl_2_ solution, 2.5 µl test DNA and 1.65 µl water were required. PCR was performed (5 min 95 °C; 10 cycles: 30 s 95 °C, 2 min 58 °C, 40 s 70 °C; 20 cycles: 25 s 95 °C, 40 s 53 °C, 40 s 70 °C; 8 min 70 °C) in a personal cycler (PE 9600, Perkin Elmer, Weiterstadt, Germany). The hybridization includes the following steps: chemical denaturation of the amplification products, hybridization of the single-stranded, biotin-labelled amplicons to membrane-bound probes, stringent washing, addition of a strepavidin/alkaline phosphatase conjugate, and an alkaline phosphatase-mediated staining reaction. The presence of the bacterial DNA was determined on a strip visually by means of coloured bands. In order to validate the correct performance of the test and the proper functioning of reagents, each strip included two control zones: Firstly, a conjugate control zone to check the binding of the conjugate on the strip and a correct chromogenic reaction, secondly an amplification control zone to check whether a control amplicon generated during amplification bound on the strip. The detection limit for all bacteria was 10^4^ genome equivalents with the exception of *A. actinomycetemcomitans* with 10^3^ genome equivalents.

### Determination of anti-CCP and anti-CEP-1

Plasma anti-CCP and anti-CEP-1 concentrations were determined via an enzyme linked immunsorbent assay (ELISA) (Euroimmun, Lübeck, Germany). Microtiter plates coated with either CCP or CEP-1 were incubated for 60 min with plasma samples (1:101 dilution) before washing. As detection antibody an anti-human IgG peroxidase-conjugate was applied for 30 min, for visualisation tetramethylbenzidine for 30 min. Optical density (OD) was determined at 450 nm (reference wavelength 620 nm). All procedures were carried out at room temperature. Antibody concentrations were determined in duplicates and mean values were calculated. The cut-off values were 5 RE/ml for anti-CCP and 20 RE/ml for anti-CEP-1. If a study participant had an antibody concentration about the cut-off he was considered as anti-CEP-1 or anti-CCP positive.

### Genomic HLA typing

Preparation of genomic DNA from fresh human venous EDTA-blood was carried out by using a salting out procedure to extract DNA from human nucleated cells [27]. After lysis of erythrocytes by Red Cell Lysis Buffer (RCLB) and protein digestion by proteinase K, DNA was extracted by precipitation of proteins using a saturating salt solution. Final purification of the DNA was effected by ethanol precipitation.

All patients were DNA typed by standard PCR (2 min 94 °C; 10 cycles: 10 s 94 °C, 1 min 65 °C; 20 cycles: 10 s 94 °C, 50 s 65 °C, 30 s 94 °C) with sequence specific primers (SSP) and by use of a thermocycler (PE 9600, Perkin Elmer, Weiterstadt, Germany) for HLA-A, -B, -C (Deutsche Dynal AG, Hamburg, and Cyclerplate System, Protrans, Hockenheim, Germany) and HLA-DRB1, -DRB3/4/5, -DQB1 allele groups (Histotype-DR,-DQ, BAG, Lich and HLA-Ready Gene DRDQLow Inno-Train, Kronberg, Germany) in low resolution technique according to the protocol provided by the manufacturer. After separation of the PCR products by electrophoresis in agarose gel containing ethidium bromide, the genotype-specific pattern became visible on a cross linker (Gel Print 1000i+, MWG Biotech, Ebersberg, Germany) by ultraviolet stimulation.

### Statistical analysis

Statistical analyses were carried out using commercially (SPSS v.19.0 package, IBM, Chicago, IL, USA) available software. Values of p ≤0.05 were considered to be significant.

Metric demographic and clinical data were checked for normal distribution using the Kolmogorov–Smirnov test and the Shapiro–Wilk test. As all metric values were not normally distributed, they were plotted as median and 25th/75th percentiles. For statistical evaluation, the Mann–Whitney U test was used.

Categorical variables such as number of patients who were positive for anti-CCP or anti-CEP-1 antibodies in dependence on periodontal diagnosis or in dependence on the occurrence of certain bacteria in subgingival plaque specimens were compared with Chi square test. The P values were corrected with Yates continuity correction. If the expected number n in one group was <5, Fisher’s exact test (p_F_) was used.

Study participants who were positive for anti-CCP or anti-CEP-1 antibodies were investigated regarding the occurrence of common HLA markers or HLA combinations. If such common HLA associations were found, we compared these results with study participants who were anti-CCP or anti-CEP-1 negative. This comparison was also carried out with Chi square test. The p values were corrected with Fisher`s exact test (p_F_).

## Results

### Demographic and clinical variables

At the time of examination, patients with GAgP were significantly younger and patients with GChP significantly older than individuals who had no periodontitis. Moreover, with the exception of the API and the number of missing teeth, all clinical periodontal parameters, such as BOP, PD, AL and percentage of teeth with AL >6 mm, were significantly increased in the two periodontitis groups as compared to the control group. Apart from *T. forsythia* in patients with GAgP all investigated periodontal bacteria were more often found in patients with GAgP and GChP in comparison to controls (Table 1).

### Anti-CCP and Anti-CEP-1 plasma level in dependence on the periodontal diagnosis

The number of individuals presenting with anti-CCP and anti-CEP-1 antibodies as well as the median plasma levels should be compared between patients with GAgP and non-periodontitis controls and between GChP and controls, respectively.

Neither in the patient groups nor in the control group anti-CCP positive individuals were found. Because the results for anti-CCP plasma concentrations below the cut-off are uncertain, we did not compare anti-CCP median levels in dependence on periodontal diagnosis, infection with certain bacteria or individual expression of HLA alleles.

For anti-CEP-1, two patients with GAgP (3.9 %) and two control individuals (2.2 %) were found positive (p_F_ = 0.622), while none of the GChP patients had anti-CEP-1 antibodies (p_F_ = 0.536).

**Fig. 1:**
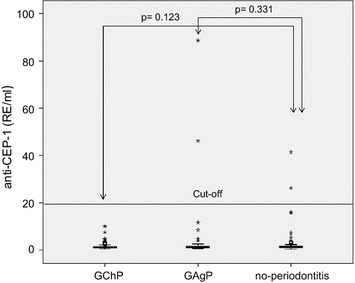
*Boxplots* of anti-CEP-1 plasma levels in dependence on periodontal diagnosis. Two patients with aggressive periodontitis (GAgP) and two controls were anti-CEP-1 positive. The medians of anti-CEP-1 plasma levels between the groups were compared using Mann–Whitney U test.

The medians of anti-CEP-1 levels between the patient groups (GAgP = 1.23 UE/ml, GChP = 1.21 UE/ml) and the controls without periodontitis (1.39 UE/ml) were not significantly different (Fig. 1).

### Anti-CEP-1 plasma level in dependence on the detection of periodontal bacteria in the subgingival plaque

**Fig. 2:**
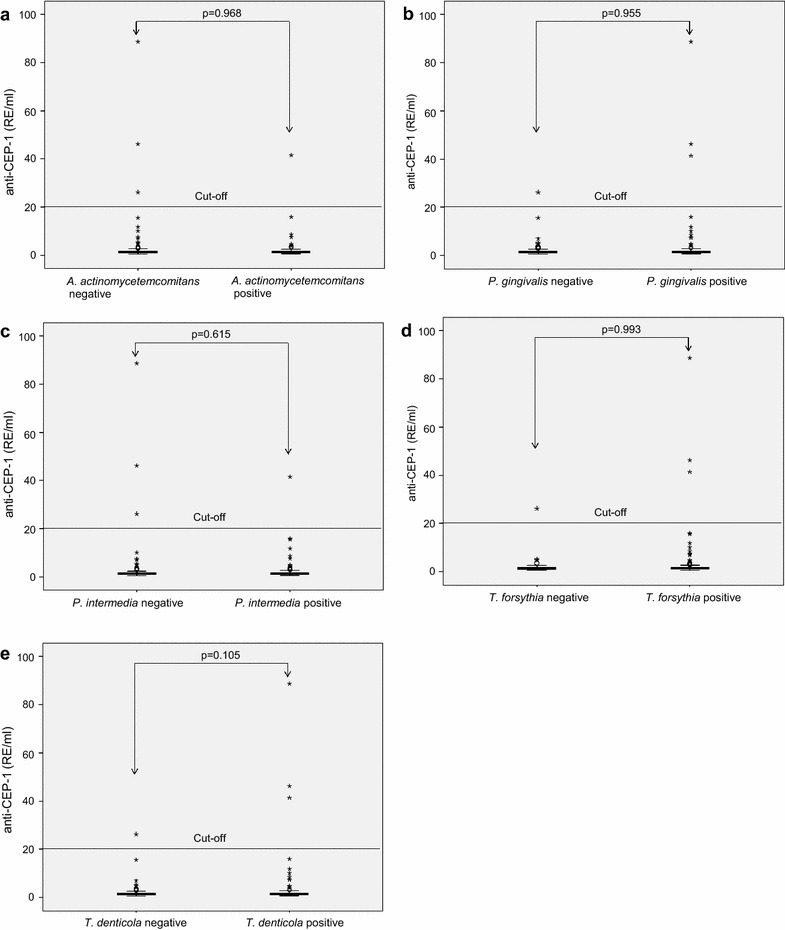
**a**–**e** *Boxplots* of anti-CEP-1 plasma levels in dependence on infection with the periodontal bacteria *A. actinomycetemcomitans* (**a**), *P. gingivalis* (**b**), *P. intermedia* (**c**), *T. forsythia* (**d**) and *T. denticola* (**e**). The medians of anti-CEP-1 plasma levels between the groups were compared using Mann–Whitney U test.

In the entire study group individuals who were infected with *P. gingivalis* were slightly more often anti-CEP-1 positive (3.2 vs. 1.1 %, p_F_ = 0.303) than study participants without *P. gingivalis* infection. In contrast, infection with *A. actinomycetemcomitans* (1.9 vs. 2.2 %)*, P. intermedia* (1.2 vs. 2.9 %), *T. forsythia* (2.0 vs. 2.6 %) or *T. denticola* (2.1 vs. 2.3 %) was associated with a slightly lower percentage of anti-CEP-1 positive individuals. These differences were not significant. Additionally, there were no significant differences in the anti-CEP-1 medians in dependence on infection with certain periodontopathic bacteria (Fig. 2a–e).

### Anti-CEP-1 plasma level in dependence on the expression of certain HLA alleles

**Fig. 3:**
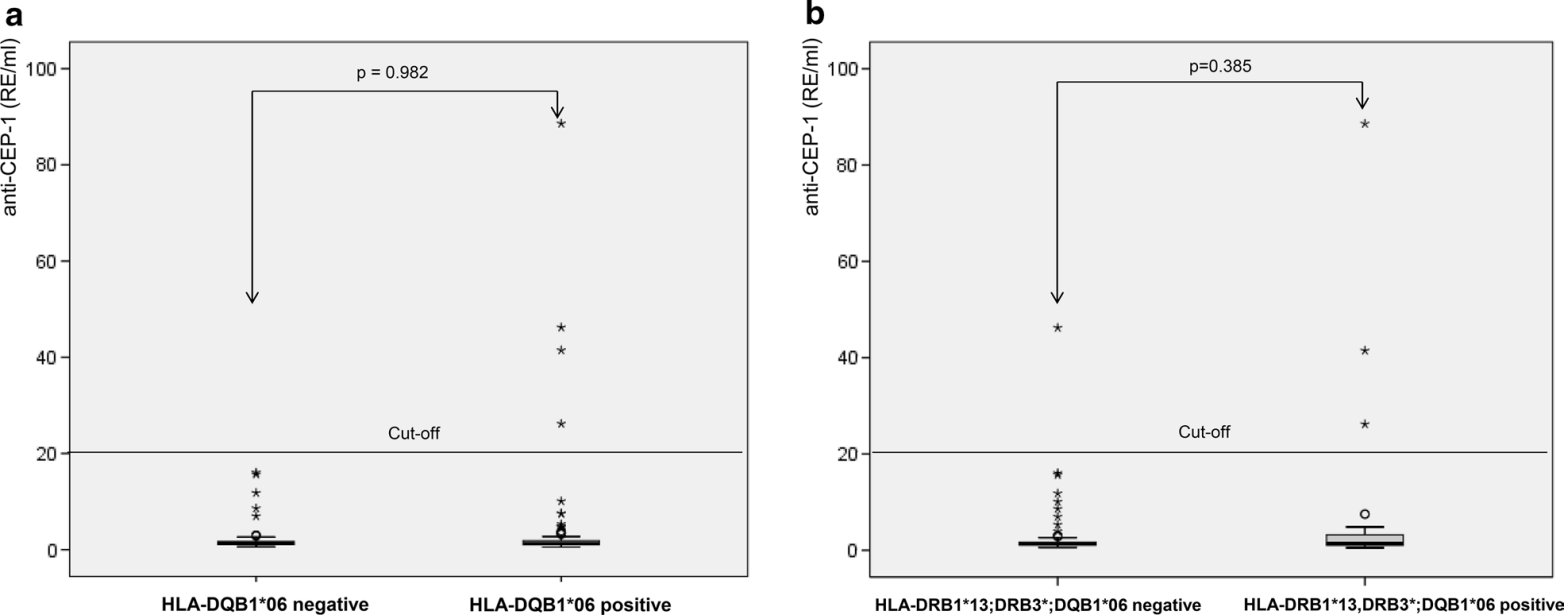
**a**, **b** *Boxplots* of anti-CEP-1 plasma levels in dependence on expression of HLA-DQB1*06 (**a**) and HLA-DRB1*13; DRB3*; DQB1*06 (**b**). The medians of anti-CEP-1 plasma levels between the groups were compared using Mann–Whitney U test.

Study participants who were carrier of the HLA marker DQB1*06 or the combination HLA-DRB1*13; DRB3*; DQB1*06 were slightly more often anti-CEP-1 positive than individuals who did not express these HLA alleles (4.43 vs. 0 %, p_F_ = 0.053 and 6.1 vs. 0.7 %, p_F_ = 0.053). The medians of the anti-CEP-1 plasma levels were not significantly different (Fig. 3a, b). There was no significant association between anti-CEP-1 levels and RA-related HLA alleles such as HLA-DRB1*01 or HLA-DRB1*04.

### Anti-CEP-1 plasma level in dependence on the smoking status

Study participants who smoked (current and past smoker) were slightly more often anti-CEP-1 positive than individuals who never smoked (3.7 vs. 0.9 %, p_F_ = 0.221). Moreover there was no significant difference in anti-CEP-1 plasma levels regarding the smoking status (1.32 vs. 1.31 RE/ml, p = 0.948).

## Discussion

Numerous studies reported that periodontitis could be a putative risk factor for RA. This relationship could be caused by (1) the coexistence of common risk factors for the two diseases such as age, smoking, and gender, (2) a common immunoregulatory imbalance [28], (3) underlying shared genetic risk factors [29], and (4) the possibility that periodontopathic bacteria could contribute to the etiology of rheumatic diseases [30]. The latter aspect was emphasized by several studies: For instance, antibodies against periodontopathogens were detected significantly more often in both serum [31–34] and synovial fluid [35] in patients with RA than in controls. Moreover, patients with periodontitis who were rheumatoid factor (RF)-seropositive presented elevated immunoglobulin (Ig)-M and IgG profiles against certain periodontophathic species versus RF-negative periodontitis subjects [36]. DNA of periodontopathic bacteria was detected in synovial fluid and the detection frequency of *P. gingivalis* was higher in patients with RA than in controls [37–39]. In in vitro experiments, *P. gingivalis* was found to invade primary chondrocytes which were isolated from knee joints and to induce cellular effects such as increasing cell apoptosis [40, 41]. These latter results might indicate that articular tissues could be damaged directly by living periodontal bacteria.

Recent studies suggest that periodontitis or the infection with *P. gingivalis* could trigger the break of immunotolerance to citrullinated peptides and in consequence the development of RA. In line with this hypothesis periodontitis was found associated with increased ACPA levels in gingival crevicular fluid [16] and peripheral blood stream [5, 17–19]. The aims of the present study were to investigate whether the generation of ACPA is different in patients with GAgP and GChP in comparison to non-periodontitis controls, whether other key periodontopathic bacteria than *P. gingivalis* are associated to increased ACPA levels at all and whether RA-related HLA alleles are associated with the amount of circulating ACPAs.

According to anamnesis no study participant suffered from RA because RA is highly associated to ACPA positivity and the aim was to investigate a putative association between periodontitis and the presence of ACPA. Of course it is not impossible that a patient with periodontitis or a control individual had a not yet discovered RA. Because the overall prevalence of RA is about 1 %, this risk is very low and only marginally affects the study results.

Among patients with GAgP we revealed slightly more patients who were positive for anti-CEP-1 when compared to controls without periodontitis (3.9 vs. 2.2 %) whereas in the GChP group no patient was anti-CEP-1 positive. Against that none of the study participants was anti-CCP positive. Among all study participants *P. gingivalis* positive patients had only slightly higher anti-CEP-1 levels (3.2 vs. 1.1 %). An infection with *A. actinomycetemcomitans*, *P. intermedia*, *T. forsythia*, and *T. denticola* was not associated with anti-CEP-1 antibodies. Individuals who were carriers of HLA-DQB1*06 or HLA-DRB1*13;DRB3*;DQB1*06 were slightly more often anti-CEP-1 positive (4.4 vs. 0 % and 6.1 vs. 0.7 %). An association to RA-associated HLA alleles could not be demonstrated.

Since our results were statistically not significant we cannot confirm the findings of three previous studies where significant associations of periodontitis or detection of *P. gingivalis* to anti-CCP and/or anti-CEP-1 levels were shown [5, 19, 20]. The percentage of anti-CEP-1 positive individuals among our patients with GAgP (3.2 %) was only slightly elevated in comparison to the normal population (2 %) [4]. Since no study individual was anti-CCP positive, our data are even below the normal distribution (asymptomatic blood donors, 0.5 % [21]). In consequence the results of the present study did not support the hypothesis of periodontitis-induced RA-associated ACPA which could predispose individuals for the development of RA.

The differences between the results revealed in our study in comparison to previous papers in which significant findings were presented can be explained as follows: First of all, ACPAs are in general highly specific for RA and occurred only rarely in non-RA groups. Although significant, the previously published differences between patients with periodontitis and non-periodontitis individuals were rather low. For instance, Lappin et al. [19] investigated 39 patients with periodontitis and 36 controls and only 3 patients with periodontitis (7.7 %) but no control individuals were anti-CCP positive. A second reason could be the different definition of patient groups. De Pablo et al. [5] examined patients with moderate and advanced periodontitis and like in Lappin et al. no distinction between ChP and AgP was made. Although our work revealed not significant results it is not excluded that ChP and AgP differ in the formation of ACPA. Thirdly, the detection limits for ACPA could be different between the various papers. For instance, the cut-off for detection of anti-CCP was 4.5 UE/ml in a Japanese study [20] in comparison to our study with a cut off of 5.0 UE/ml. Fourthly, the amount of smokers may influence the number of ACPA-positive patients and controls [6]. Lappin et al. [19] reported about a higher prevalence of smokers among patients with periodontitis (41 %) and controls (44 %) compared to Pablo et al. [5] (24 vs. 22 %) and our study (GAgP 33 %, GChP 27.1 %, controls 21.3 %). However, regarding our data we could not show an association between smoking status and anti-CEP-1 antibodies. Fifthly, studies revealed that the generation of autoantibodies is influenced by the individual expression of certain RA-associated HLA-DRB1 molecules [13, 42]. Although we were unable to show associations of HLA-DRB1*01 and HLA-DRB1*04 to both anti-CCP and anti-CEP-1 levels, the HLA genetic background may be important to initiate an autoimmune reaction to citrullinated peptides. Since the distribution of HLA alleles depends on the ethnic origin, differences in ACPA levels in various groups are probable.

The revealed associations of HLA-DQB1*06 and HLA-DRB1*13;DRB3*;DQB1*06 to anti-CEP-1 have to be interpreted with caution due to the small number of anti-CEP-1 positive individuals in the study. Moreover, a simultaneous association of these HLA alleles to GAgP could not be observed. Whether these HLA alleles are actually important for the binding and presentation of citrullinated alpha-enolase needs to be investigated in HLA binding assays.

In summary we did not find significant associations to peripheral anti-CCP and anti-CEP-1 antibodies in both patients with GAgP and GChP in comparison to non-periodontitis controls. Patients who were infected with *P. gingivalis* had only slightly elevated anti-CEP-1 levels. None of the other investigated bacteria were significantly associated with circulating ACPA. Carriers of HLA-DQB1*06 or HLA-DRB1*13;DRB3*;DQB1*06 had slightly higher anti-CEP-1 levels than study participants who were not carrier of these HLA alleles. An association of ACPA to RA linked HLA-alleles could not be confirmed.

## Conclusion

Both, GAgP and GChP and an infection with *P. gingivalis*, *A. actinomycetemcomitans*, *P. intermedia*, *T. forsythia*, and *T. denticola* were not significantly associated to peripheral anti-CCP and anti-CEP-1 antibodies. Moreover, a significant association of HLA to ACPA could not be detected. The underlying biological mechanisms for the relationship between periodontitis and RA need to be investigated in further studies.
